# Cartilage degradation is followed by PAC1 receptor reduction in articular cartilage of human knee joints

**DOI:** 10.1007/s11357-025-01689-4

**Published:** 2025-05-14

**Authors:** Kálmán Rácz, Yonatan Segal, Kinga Lénárt, Csaba Fillér, Anna Tóth, Vince Szegeczki, Péter Gergely, Róza Zákány, Dóra Reglődi, Tamás Juhász

**Affiliations:** 1 Department of Forensic Medicine, Faculty of Medicine, University of Debrecen, Nagyerdei Krt. 98, 4032 Debrecen, Hungary; 2 Department of Anatomy, Histology and Embryology, Faculty of Medicine, University of Debrecen, Nagyerdei Krt. 98, 4032 Debrecen, Hungary; 3 Department of Anatomy, Medical School, HUN-REN-PTE PACAP Research Team, University of Pécs, Szigeti Út 12, 7624 Pécs, Hungary

**Keywords:** Cartilage thickness, PAC1 receptor, Cartilage degradation, Sox9, Osteoarthritis

## Abstract

Pituitary adenylate cyclase activating polypeptide (PACAP) is a neuropeptide expressed in the nervous system and also in various peripheral tissues, including the musculoskeletal system. PACAP has an important function in the regulation of chondrogenesis and plays a protective role in cartilage oxidative and mechanical stress. PACAP knockout (KO) mice show early signs of aging and osteoarthritis in knee joint articular cartilage. Its specific, most potent receptor is the PAC1 receptor, the activation of which leads to enhanced Sox9 expression and subsequently, it increases the expression of collagen type II, glucosaminoglycans and aggrecan. In the present study, we investigated articular cartilage of human knee joints taken from cadavers of varying ages. Thickness and extracellular matrix content of articular cartilage of knee joints decreases with aging. The cartilage degeneration process most likely begins between the ages of 40 to 50. Expression of PAC1 receptor decreases in parallel with the reduction of cartilage thickness, leading to subsequent reduced Sox9 expression with cartilage specific matrix production. In summary, we found correlation in the reduction of cartilage thickness and quality together with PAC1 receptor expression and activity.

**Supplementary Information:**

The online version contains supplementary material available at 10.1007/s11357-025-01689-4.

## Introduction

Knee joint is the largest and most complex synovial joint of the human body covered by articular cartilage, the surface of which allows smooth movement in the synovial joints and distributes forces to the subchondral bone during locomotion. Articular cartilage is a specialized avascular and aneural connective tissue consisting of extensive extracellular matrix and chondrocytes which maintain tissue integrity by producing the extracellular matrix (ECM) components. On the other hand, the activation of chondrocytes and regeneration capability of cartilage matrix is very slow if it happens at all [1, 2]. Articular cartilage matrix is identified by collagen type II, glycosaminoglycans (GAGs), proteoglycans, and multi-adhesive glycoproteins, the specific architecture of which is crucial for chondrocyte survival. Around chondrocytes are tightly intertwined collagen fibers, almost exclusively type VI, forming the pericellular matrix, while the territorial matrix is a region that resides in a more distant location from the chondrocytes. This region consists of a randomly arranged network of collagen type II fibrils with a lesser quantity of other types of collagen such as type IX collagen [3]. Moreover, it has lesser amount of sulphated proteoglycan and does not stain as strongly as the capsular matrix. The interterritorial matrix fills the space between chondrocyte groups found around the territorial matrix. With its high water content and the specific collagen fiber orientation pattern, this tissue is responsible for the weight-bearing capacity and flexibility of the joint [4].

Throughout adulthood, cartilage remains a remnant of the original hyaline cartilage templates for growing bones and stays throughout adult life [5, 6]. Adult articular cartilage has a thickness of 2–5 mm and is divided into four zones. The superficial (tangential) zone lies closest to the articular surface and is pressure resistant. Several elongated and flattened chondrocytes are clustered in fascicles parallel to the free surface and surrounded by fibrils of type II collagen. Intermediate (transitional) zone is located beneath the superficial zone and contains round chondrocytes wholly dispersed throughout the matrix. Collagen fibrils are loosely arranged on the surface and oriented in some way obliquely to the cartilage surface. Deep (radial) zone contains small, round chondrocytes which are arranged in small columns like an array perpendicularly to the cartilage surface. Between the columns of the bone run collagen fibrils parallel to the bone’s long axis. Calcified zone consists of a calcified matrix with small chondrocytes. Tidemark is a smooth, undulating, heavy-calcified line separating the deep (radial) zone from it [7, 8]. Up to this line, chondrocytes proliferate within the cartilage lacunae that provide new cells for interstitial growth. Chondrocytes slowly migrate from this area toward the joint surface during potential cartilage regeneration [8, 9]. Mature articular cartilage undergoes a very slow renewal process. Slow growth can be attributed to type II collagen’s highly stable network and its long half-life proteoglycan molecules. Additionally, the activity of metalloproteinases (MMP1 and MMP13) is low in healthy articular cartilage [10]. In pathological alterations, such as inflammation, oxidative stress, or damaging mechanical force, the composition of cartilage-specific ECM can be disintegrated and cartilage degeneration may occur [11]. Osteoarthritis (OA), also known as degenerative joint disease, is a leading cause of disability worldwide. Due to the aging of the population and the obesity epidemic, the number of people suffering from symptomatic knee osteoarthritis is likely to increase further [12]. Although the pathophysiology of the disease is not fully understood and is still under investigation, it is acknowledged that knee OA has multiple factors that can lead to the condition, and aging increases the risk of its formation [13].

Production of cartilage-specific ECM components is regulated by various and complex signaling cascades. Although several elements of matrix-secreting genes are known, major components of possible regeneration processes are still unknown. The classical signaling pathways of matrix production is initiated by PKA activation [14, 15] which can phosphorylate several transcription factors, such as Sox9 or CREB [16], and via these PKA can regulate collagen type II and aggrecan production. On the other hand, hedgehog signaling, ERK signalization, or other calcium-dependent cascades are also involved in proper matrix production and regulation of physiological orientation [15, 17, 18]. Several new cartilage-regulating elements, previously related mostly to excitable cells, have been identified in the last decades such as NMDA [19], TRPV receptors [20], sodium- and potassium-activated channels [21] or PACAP signalization [22]. However, their function in pathological disorders, aging, or in regeneration of human articular cartilage is not clarified in detail.

Pituitary adenylate cyclase-activating polypeptide (PACAP) is a neurohormone with 38 amino acid sequence. It was first isolated from the hypothalamus and identified with its abilities to stimulate an intracellular signaling cascade, that begins with the adenylate cyclase (AC) enzyme-converting adenosine triphosphate (ATP) to cyclic adenosine monophosphate (cAMP), upon interaction with its appropriate receptors on the cell membrane [23]. Later, the neurotransmitter PACAP was found to be widely expressed in multiple regions of the brain as well as in various peripheral tissues all over the body [24]. There have been three PACAP receptors identified in vertebrates such as PAC1-R, that binds PACAP with high affinity and VPAC1-R, VPAC2-R, that are equally sensitive to PACAP, and VIP [25]. The activation of these receptors triggers AC activity leading to increased concentration of intracellular cAMP, activating PKA signaling subsequently [26]. Cartilage-protecting role of PACAP signalization has been shown in in vitro oxidative stress in cartilage formation [22] or in increased mechanical stress in vitro [22, 27]. Furthermore, PACAP can decrease the activity of matrix-degrading enzymes in vitro [28, 29]. It has been published that knee joints of PACAP knockout (KO) mice show early signs of osteoarthritis during aging [30], and PACAP receptor expression has been shown in healthy and osteoarthritic cartilage [31]. PACAP plays a fundamental role in development and regeneration of other skeletal element such as bone and callus formation [32, 33] or tooth development [34, 35].

It is well known that aging of articular cartilage can result in a serious degradation of cartilage matrix and can induce osteoarthritis [36]. Pathological identification of chondropathies and osteoarthritis is well described, but the exact process of aging-related formation of pathological disorders are not yet clarified [37]. Chondrocytes of articular cartilage have long lifespan but little is known about their signaling alterations or their possible reactivation in aging. Based on our previous results showing that the lack of PACAP resulted in the degradation of articular cartilage in PACAP KO mice, it raises an interesting question about the potential role of the neuropeptide in the aging process of human cartilage. Therefore, we investigated human articular cartilage from knee joints to identify a correlation between the aging of cartilage and the expression levels of the receptors involved in PACAP signaling (PAC1-R, VPAC1-R and VPAC2-R), along with other parameters indicating degeneration of the cartilage tissue.

## Material and methods

### Human articular cartilage samples

Samples of articular cartilage were collected from the right medial condyle of femur, removed from recently died individuals. Different groups were established based on ages of individual in decades (ethical num.: 28,996–2/2018/EKU). We accepted samples maximum 9 days postmortem, as cartilage degradation in cadavers starts after this period, and all individuals were selected out with diagnosed pathological cartilage disorders [38]. After obtaining the samples, articular cartilages were chopped into two pieces for histological analysis, and the articular cartilage and subchondral structures were dissected from the bone, using a surgical scalpel for Western blot analysis.

### Histological analysis

Samples were washed in PBS (phosphate buffer solution) three times and fixed in 10% formalin fixative for 72 h. Bones were decalcified in 4% EDTA (Sigma-Aldrich, MO, USA) for 4 weeks until the bones and cartilage tissue became soft. Afterwards, the decalcifying solution was washed out with PBS for 30 min, and samples were embedded in paraffin. Serial sections of 7-µm thick slides were prepared with a rotation microtome (Leica, Wetzlar, Germany). The samples were stained with hematoxylin and eosin (HE) staining (Sigma-Aldrich, MO, USA), dimethyl-methylene blue (DMMB) staining (Sigma-Aldrich, MO, USA), and picrosirius red staining (Sigma-Aldrich, MO, USA) according to the instructions of the manufacturer. At the end of the staining process, the samples were covered with DPX (Sigma-Aldrich, MO, USA). Histological slides stained with HE and DMMB were examined with a light microscope BX53 Olympus (Olympus, Tokyo, Japan) with constant camera and exposure settings.

### Polarization light microscopy

The samples stained with picrosirius red were examined by polarization lens where the light plane filter was turned with λ/4 with constant camera and exposure settings. Two photos were taken, first under normal light and then in polarized light. A-1 mm wide region of interest extending from the cartilage surface to cartilage-bone interface was used to analyze the images. Semiquantitative polarization light microscopy (PLM) scoring system was used where scores range between 0 and 5, with lower score indicating degrading cartilage; a score of 0 describes cartilage specimens that have sparse patches of birefringence that are neither parallel nor perpendicularly orientated indicating disorganized cartilage. A score of 5 indicates normal adult hyaline cartilage, where a distinct and smooth superficial zone is present. Birefringence of collagen shows both parallel and perpendicularly aligned orientations in deeper zones. [39]. Superficial, intermediate, and deeps zones were investigated separately. Furthermore, the pixel intensity of green (thin) and red (thick) collagen fibers was also measured by ImageJ 1.40 g freeware, and semiquantitative data were given and normalized to the data of group 0–10.

### Thickness measurement of articular cartilage

For the measurement of articular cartilage thickness, a customized mathematical formula was used as we described previously [30] on HE stained slides, under four times magnification objective at least in five independent samples in every age group. Ten individual measurements were performed on each slide. Similar measurements were done on DMMB stained slides where only the thickness of metachromatically stained area was measured.

### OA grading (OARSI grading system)

The OARSI (Osteoarthritis Research Society International) grading system is a commonly used tool for evaluating the severity of OA in articular cartilage. It offers a standardized approach for assessing histological changes in cartilage. OA that involves deeper cartilage is therefore assumed to be an advanced form of the disease and is a good indicator of progression. An OA score is derived from the combination of OA stage and OA grade. As the cartilage erosion progresses, the adjacent bone becomes the articular surface. Grading OA severity scaled to 1–6 grades while normal cartilage is grade 0. Histopathological scoring was done by a pathologist in five independent samples in every age group according to OARSI grading system described previously [40].

### Immunohistochemistry

The localization of PAC1-R was followed with immunohistochemistry using a polyclonal antibody (Sigma-Aldrich MO, USA, ADYCAP R1). After deparaffinization in descending alcohol row and washing in PBST (phosphate-buffered saline supplemented with 1% Tween-20) three times, unspecific binding sites were blocked with bovine serum albumin (BSA) (Amresco, CA, USA) at 37 ℃ for 30 min, then slides were washed in PBS 3 × 10 min. Primary antibody of PAC1-R in dilution of 1:500 was used overnight at 4 ℃. After washing in PBS secondary antibody, anti-rabbit-Alexa555 in dilution 1:1000 (Invitrogen, MA, USA) was applied. For nuclear visualization, slides were covered with DAPI (Vector Laboratories, CA, USA). Fluorescent images were taken with an Olympus FV1000S confocal microscope (Olympus Co., Tokyo, Japan) using × 60 oil immersion objective (NA: 1.3). For excitation, a laser line of 543 nm was used. The average pixel time was 4 μs. Z stack image series of 1-μm optical thickness were recorded in sequential scan mode with constant settings. Images of Alexa555 and DAPI were overlaid using Adobe Photoshop version 10.0 software. For negative control, PAC1-R KO mouse samples were used (Supplementary Fig. 1).

### Western blot

Parts of the articular cartilage were inserted into Eppendorf tubes, and 100 μL of PIC (Aprotinin (10 ug/mL), 5 mM Benzamidine, Leupeptin (10 ug/mL), Trypsine inhibitor (10 ug/mL), 1 mM PMSF, 5 mM EDTA, 1 mM EGTA, 8 mM Na-Fluoride, 1 mM Na-orthovanadate) (Sigma-Aldrich, MO, USA) was added to samples and kept at a temperature of − 80 ℃. The CG-200 Freezer/Mill was used as a cryogenic mill that precools in liquid nitrogen and grinds samples (Cole-Parmer, Illinois, USA) for 30 s till it became a fine powder. After adding 100 μL of PIC, suspensions were sonicated by pulsing burst for 30 s at 40 A (Cole-Parmer, Illinois, USA). For Western blotting, total tissue lysates were used. Samples for SDS-PAGE were prepared by the addition of Laemmli electrophoresis sample buffer (4% SDS, 10% 2-mercaptoethanol, 20% glycerol, 0.004% bromophenol blue, 0.125 M TrisHCl pH 6.8) to tissue lysates to set equal protein concentration of samples and boiled for 10 min. About 10 µg of protein was separated by 7.5% SDS-PAGE gel for detection of PAC1-R, VPAC1-R, VPAC2-R, Sox9, P-Sox9, and Actin. Proteins were transferred electrophoretically to nitrocellulose membranes. After blocking with 10% nonfat dry milk containing 0.1% Tween 20 (PBST), membranes were washed and exposed to the primary antibodies overnight at 4 ℃. Details of antibodies are shown in Table 1. Signals were detected by enhanced chemiluminescence (Advansta, CA, USA) according to the instructions of the manufacturer. Signals were developed in the FluorochemE gel documentary system (Protein Simple, CA, USA). Optical density of Western blot signals was measured using ImageJ 1.40 g freeware, and results were normalized to that of 0–10-year control group.

**Table 1:** Table of antibodies used in the experiments

*Antibody*	*Host animal*	*Dilution*	*Distributor*
Anti-PAC1	Rabbit, polyclonal	1:400	Sigma-Aldrich, St. Louis, MO, USA
Anti-VPAC1	Rabbit, polyclonal	1:800	Alomone Labs., Jerusalem, Israel
Anti-VPAC2	Rabbit, polyclonal	1:600	Abcam, Cambridge, UK
Anti-Sox9	Rabbit, polyclonal	1:600	Abcam, Cambridge, UK
Anti-P-Sox9	Rabbit, polyclonal	1:800	Sigma-Aldrich, St. Louis, MO, USA
Anti-Actin	Mouse, monoclonal	1:10,000	Sigma-Aldrich, St. Louis, MO, USA

### Statistical analysis

All data are representative of at least five independent experiments. Where applicable, data are expressed as mean ± SEM. Statistical analysis was performed by Student’s *t* test. The threshold for statistically significant differences as compared to respective control was set at *p < 0.05.

## Results

### General morphology of articular cartilage in aging

**Fig. 1:**
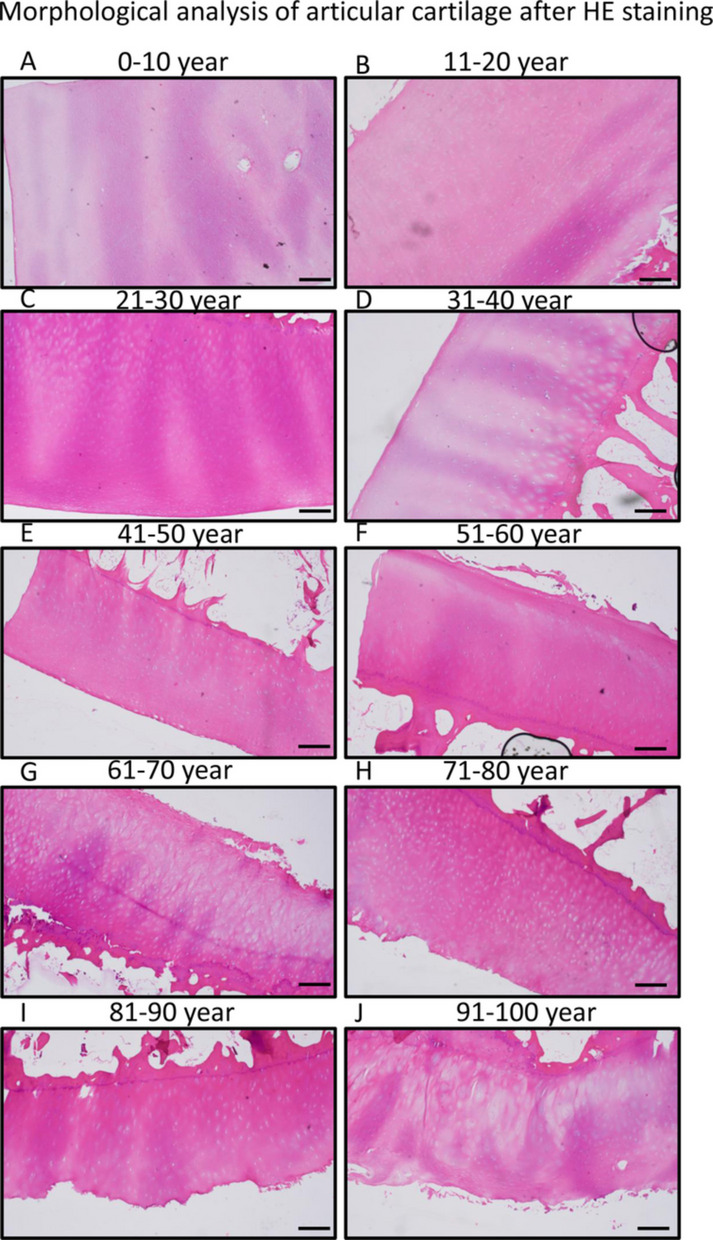
Morphological analysis of knee joints of human samples. Hematoxylin–eosin (HE) staining was used to visualize histological differences. Following groups of aging was set 0–10 age (**A**), 11–20 age (**B**), 21–30 age (**C**), 31–40 age (**D**), 41–50 age (**E**), 51–60 age (**F**), 61–70 age (**G**), 71–80 age (**H**), 81–90 age (**I**), and 91–100 (**J**). Original magnification was 4 ×. Scale bar, 2 mm. Representative data of at least five independent experiments

For general morphological analysis, HE staining was performed. Zones of articular cartilage were analyzed by investigation of extracellular matrix staining intensity and discontinuity of articular surface. In newborn babies or in childhood articular cartilage, zones were not always judgeable as enchondral ossification was still in progress (Fig. 1A). Without any pathological disorders, normal cartilage morphology was determined. In young individuals between the ages of 11–20, the formation of zones of articular cartilage was identified without any pathological disorders. Superficial zones were stained normally showing strong collagen accumulation and flattened chondrocytes in sequential row. In the intermediate zone, round shaped chondrocytes forming clusters, so called chondrons, appeared with histologically properly stained territorial and interterritorial matrix. Then chondrons organized into columns and calcified zones became visible with smaller round shape chondrocytes. Tidemark of articular cartilage was well distinguishable (Fig. 1B). In young adults aged 21–30, normal articular architecture was observed, with histologically ideal staining intensity present in various components of the ECM, including the capsular, territorial, and interterritorial matrices (Fig. 1C). The articular facets showed normal integrity without signs of discontinuity. Superficial and intermediate zones showed the characteristics of healthy hyaline cartilage. Moreover, chondrocytes were arranged into columns and became hypertrophic (Fig. 1C) in the deep zone.

In the age group 31–40, generally, the morphology of articular cartilage still showed physiological characteristics of healthy cartilage tissue (Fig. 1D). Although the staining intensity of the extracellular matrix appeared similar to that of young adults, there were some differences in capsular matrix intensity indicating the decreased matrix productive ability of chondrocytes. Weaker eosin staining of the ECM indicates lower protein content, such as collagen, which is the major component of healthy cartilage tissue. Structural disorders in superficial, intermediate, and deep zones were not identified. The surface integrity of the superficial zone did not have pathological alterations (Fig. 1D). In the age group of 41–50, staining intensity of certain matrix areas, such as territorial and interterritorial matrix, became paler and wider. Moreover, discontinuity could be identified on the surface of the articular cartilage which later turns to lesions or complete surface degradation (Fig. 1E). Deep zone of articular cartilage did not frequently show pathological alterations. In the age group of 51–60 samples, the surface discontinuity was well visible, and lesions or superficial peel off was visible (Fig. 1F). On the other hand, no significant alterations were visible in the deeper zones as the territorial and interterritorial matrix integrity was kept and hypertrophic columns were normally oriented (Fig. 1F). After the age of 61, various disorders were visualized such as superficial surface lesions or territorial and interterritorial matrix disintegrity (Fig. 1G). Similarly, in the age group of 71–80, where deeper superficial lesions and in some cases, loss of the superficial layer was identified (Fig. 1H). Lesions in some cases reached the intermediate zone, and widened deep zone was present (Fig. 1H). Further degradation was visible in age group 81–90 with similar characteristics (Fig. 1I), and complete matrix disorders and histological alterations were seen in the eldest individuals (Fig. 1J).

### Glucosaminoglycan content of articular cartilage in aging

**Fig. 2:**
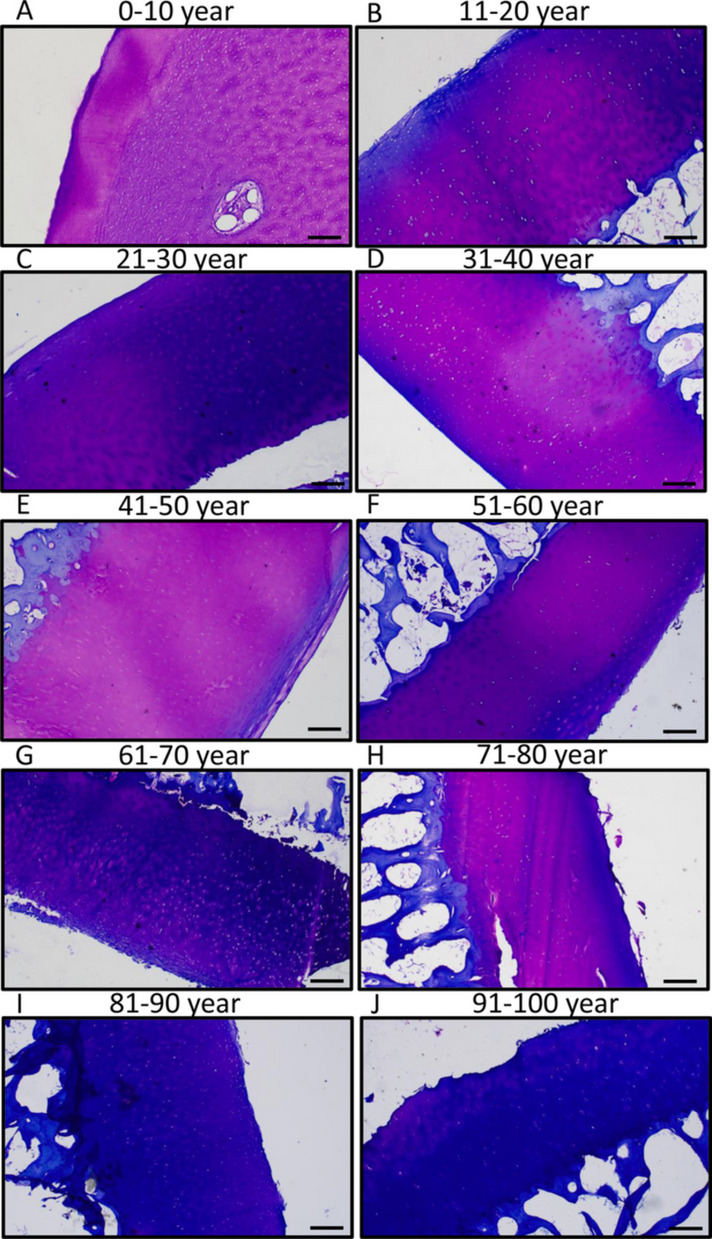
Metachromatic area analysis of knee joints of human samples. Dimethyl-methylene blue (DMMB) staining was used to visualize glycosaminoglycan expression differences. Following groups of aging was set 0–10 age (**A**), 11–20 age (**B**), 21–30 age (**C**), 31–40 age (**D**), 41–50 age (**E**), 51–60 age (**F**), 61–70 age (**G**), 71–80 age (**H**), 81–90 age (**I**), and 91–100 (**J**). Original magnification was 4 ×. Scale bar, 2 mm. Representative data of at least five independent experiments

Metachromatically stained cartilage matrix is proportional with the glucosaminoglycan content and also indicates the integrity of articular cartilage. In young individuals, the newly formed metachromatic matrix was well visible with stronger staining around the chondrons (Fig. 2A). Strong metachromatic staining was identified until the age of 40 in superficial and also in deeper zones (Fig. 2B, C, D). After the age of 41, the metachromatic staining became paler and sometimes disappeared in superficial zones of articular cartilage (Fig. 2E). Moreover, orthochromatic blue color was visible in the superficial zone (Fig. 2E). Parallel with the superficial degradation, the metachromatic color was varying in elder individuals. In some cases, it became paler, or strong orthochromatic color was detected in the complete thickness of articular cartilage (Fig. 2F-J). In the superficial lesion, orthochromatic staining was dominant even if the deeper zones had metachromatic staining (Fig. 2F-J). After the age of 80, dominantly orthochromatic staining was visible in the entire thickness of articular cartilage (Fig. 2I and J).

### Thickness of articular cartilage decreased in aging

**Fig. 3:**
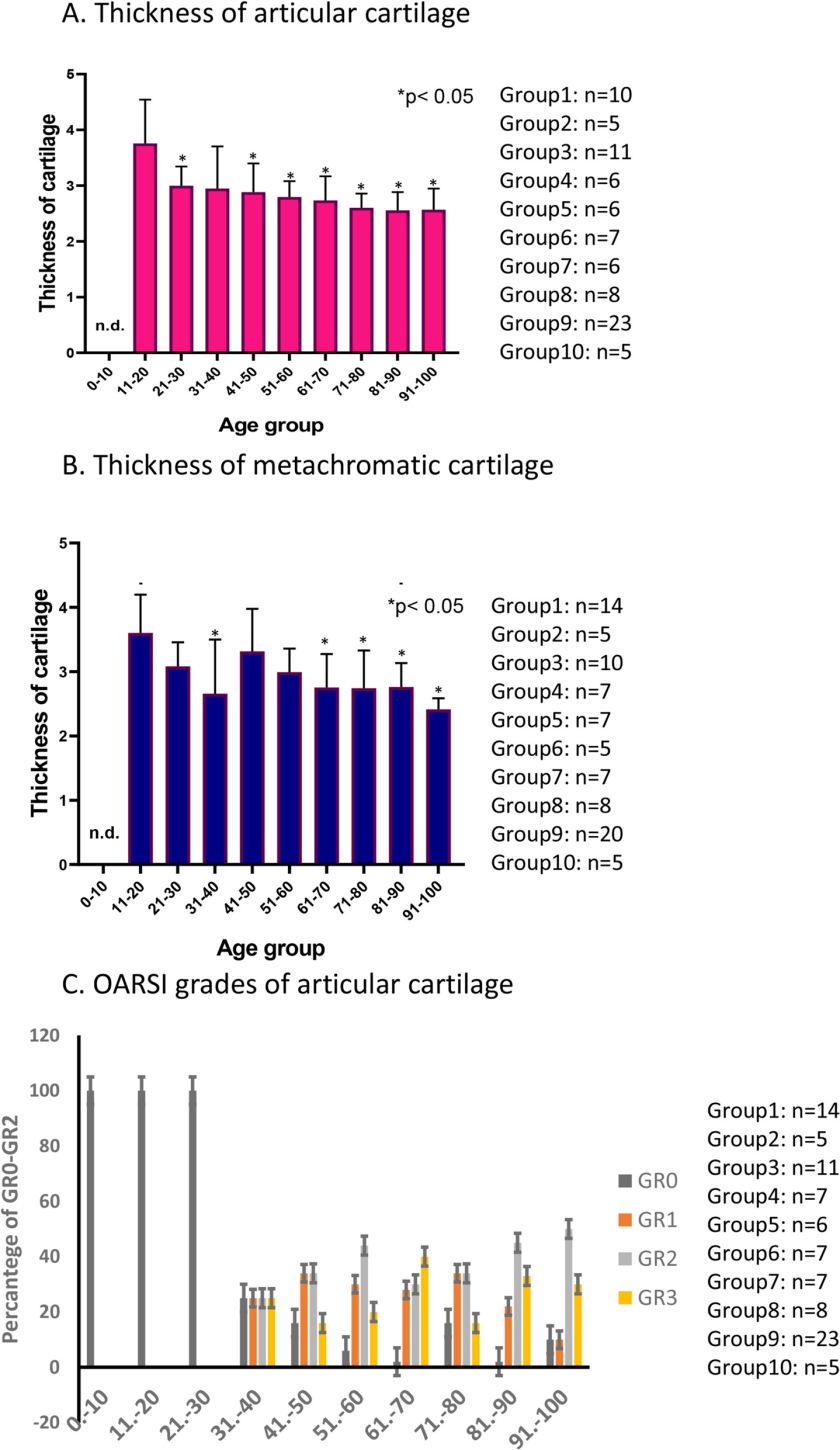
Thickness of articular cartilage. Geometric analysis of articular cartilage with HE staining (**A**) and measurement of metachromatic area with DMMB staining (**B**). Measurable number of articular cartilages shown at least *n* = 5 cases. Asterisks indicate significant (*p < 0.05) difference in thickness of cartilage compared to the respective control. OARSI scoring (**C**) according to the international standards. Grades 0, 1, 2, 3, and 4 represent the degradation level of articular cartilage

Based on the HE staining, we focused on the measurement of two parameters. The first one diagnoses the thickness of the cartilage in each decade (Fig. 3A). The thickness of articular cartilage was measured till the subchondral bone. Similarly to general morphology, in young individuals, the thickness was not measurable because of endochondral ossification (Fig. 3A). After the age of 21, a slow but gradual decrease was measured (Fig. 3A). The second parameter was based on thickness of metachromatic matrix which can better inform us about the healthy articular cartilage thickness (Fig. 3B). Similarly to the previous results, the young individuals’ metachromatically stained cartilage matrix was not determinable. Moreover, after the age of 21, it showed a continuous reduction with the exception of 41–50 age group where a slight elevation was detected (Fig. 3B). An OARSI pathological scoring was also performed to give detailed analysis of morphological differences (Fig. 3C). Until the age of 40, no pathological disorders were determined (Fig. 3C). Grade 1, 2, and 3 chondropathies appeared in the 41–50 age group, with an equal distribution among individuals (Fig. 3C). In the age group of 51–60, increased grades 1 and 2 chondropathy started to appear. This elevation was dominant during aging, and grade 3 chondropathies became stronger as individuals approached 100 years of age (Fig. 3C). Interestingly, no significant correlation was visible between aging and severe chondropathy formation; different grades occurred almost with same percentage (Fig. 3C).

### Collagen orientation and thickness in aging

The orientation of collagen type II in the superficial zone is parallel to the surface in healthy articular cartilage, then they run perpendicular to it in the intermediate zone. Furthermore, around the chondrons, the orientation of collagen type VI was circular while in the calcification zone, collagen type X started to be expressed (Fig. 8). With picrosirius red staining, collagens can be visualized without any specificity (Supplementary Fig. 2). However, by rotating the polarized light with *λ*/4, collagen orientation can be demonstrated after picrosirius red staining in polarization microscopy. Thick collagen fibers appear in light shiny red color, and thinner fibers have a light green characteristics while the regular orientation of collagen type IV around the chondrons shows a so called maltan cross [41]. In healthy adult hyaline cartilage, collagen exhibited a thin, uniform layer of birefringence at the articular surface due to parallel alignment of collagen in the superficial zone. The random arrangement of collagen type II fibrils in the intermediate zone can lead to decreased birefringence with a circular orientation around chondrons. The presence of orthogonally arranged thin collagen II fibrils in the deep zone resulted in low birefringence. During OA formation, the orientation and birefringence of collagen can alter [42, 43]. The measurement of the ratio of thick to thin collagen fibers can provide semiquantitative information about the structural integrity of collagens in the articular cartilage, while the presence of maltan crosses can serve as a good qualitative characteristic of proper cartilage ECM orientation.

**Fig. 4:**
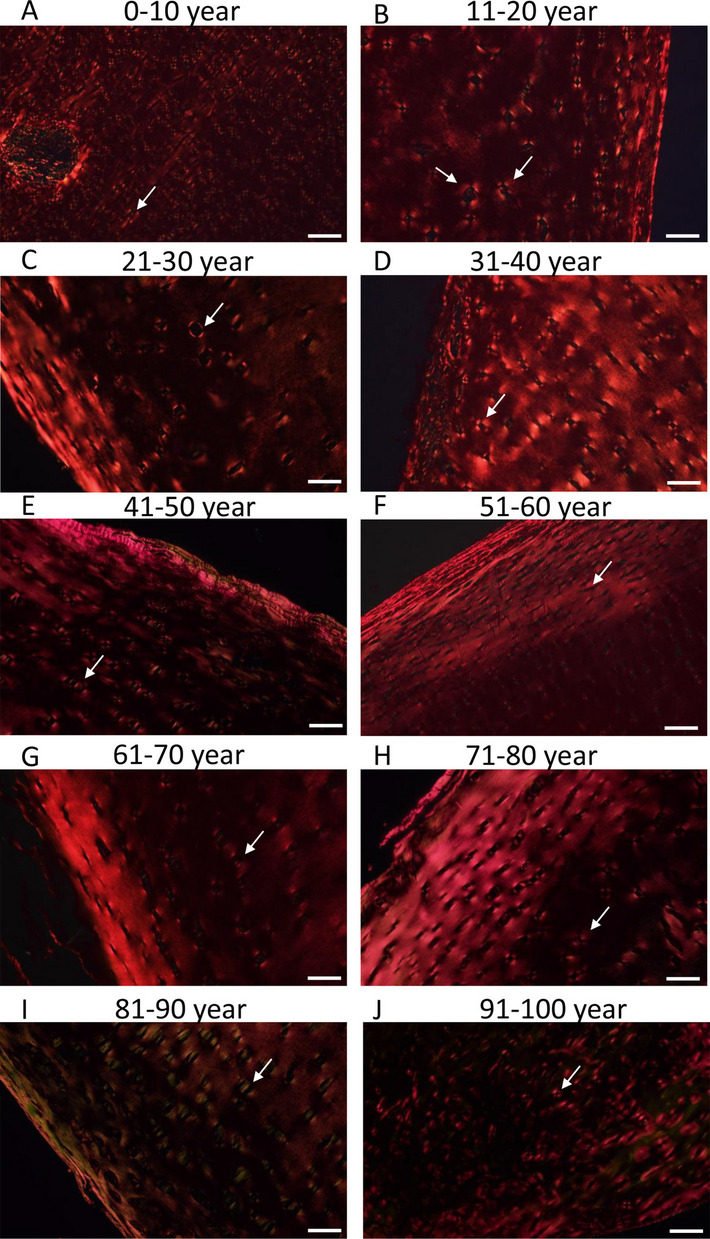
Polarization light microscopical (PLM) analysis of articular cartilage. Picrosirius red staining was used to visualize the birefringence of collagen fibrils. Following groups of aging was set 0–10 age (**A**), 11–20 age (**B**), 21–30 age (**C**), 31–40 age (**D**), 41–50 age (**E**), 51–60 age (**F**), 61–70 age (**G**), 71–80 age (**H**), 81–90 age (**I**), and 91–100 (**J**). Arrows represent the maltan crosses around chondrons. Original magnification was 10 ×. Scale bar, 500 μm. Representative data of at least five independent experiments

**Fig. 5:**
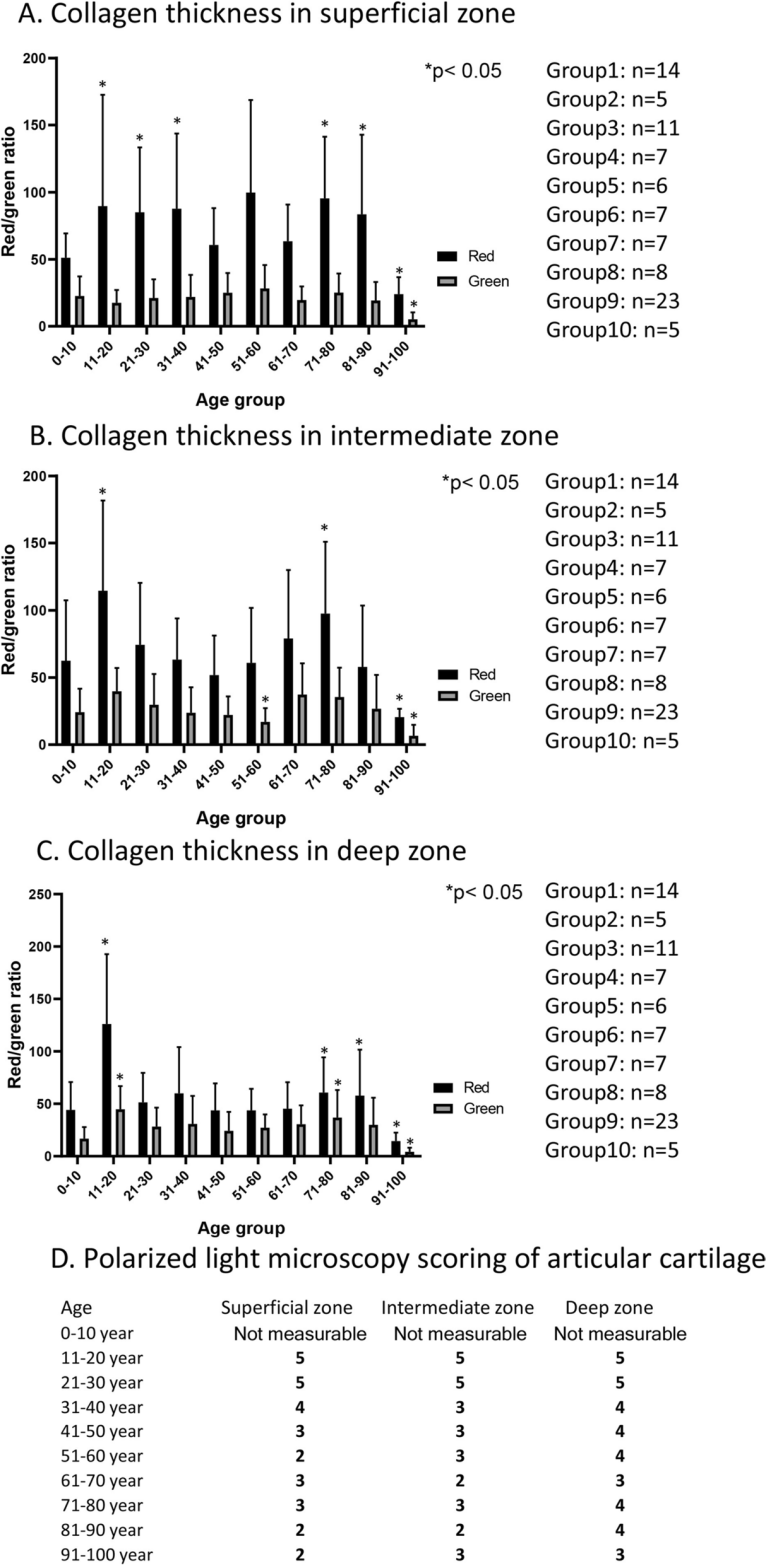
Thickness of collagen fibers in articular cartilage. Red and green pixel intensity was measured in the superficial zone (**A**), intermediate zone (**B**), and deep zone (**C**) of articular cartilage. Measurable number of articular cartilages shown at least *n* = 5 cases. Asterisks indicate significant (*p < 0.05) difference in thickness of thick and thin collagen fibers compared to the respective control. PLM scoring (D) according to the international standards. Score of PLM represents disorientation of collagen fibers in superficial, intermediate and deep zones

The zones of articular cartilage were not well distinguishable in infants; superficial, intermediate, and deep zones were barely identifiable in picrosirius red staining. In childhood, collagen type II was orientated parallel with the articular surface in a very thin layer, and numerous maltan crosses were visible in the intermediate and deep zones (Fig. 4A). As the production of ECM, especially collagens, progressed during these ages, the number of thicker collagens continuously increased, although thinner fibers could also be observed in greater amounts in all zones (Fig. 5A-C). On the other hand, polarized light microscopy (PLM) scoring cannot be defined due to the continuous reorganization of collagen matrix. In young individuals, typical histological characteristics and orientation of collagens could be visualized. A thin but characteristic superficial layer with parallelly running collagen fibers was visualized till the age of 20 (Fig. 4A and B). The number of thicker collagen fibers increased in this layer, while the proportion of thinner fibers decreased (Fig. 5A). The middle and deep layers were rich in regularly oriented maltan crosses; moreover, very high thick collagen ratio could be seen in these zones (Fig. 5B and C). In a healthy adult, the PLM score for cartilage reached 5 in all zones (Fig. 5D). In the age group of 21–30, the superficial zone thickened and exhibited a very strong appearance of thick, collagen fibers oriented parallel to the surface (Figs. 4 C and 5 A), with a PLM score of 5 (Fig. 5D). The middle and deep zones in this group also showed a great number of maltan crosses although a slight reduction in thick collagen fibers was detected (Figs. 4 C and 5B, C), with a score of 5 PLM. Interestingly, in the age group of 31–40, the superficial zone was slightly thinner, but the same number of thick and thin collagen fibers was detected (Figs. 4D and 5 A). The PLM score showed a slight reduction (Fig. 5D). On the other hand, the intermediate and deep zones started to show early signs of disintegration of maltan crosses, characterized by the appearance of wider red courts of chondrons. Moreover, further reduction was detected in the number of thick collagen fibers in these zones (Figs. 4D and 5B, C). The PLM score also indicated alterations, with a score of 3 in the intermediate zone and 4 in the deep zone of cartilage (Fig. 5D).

After the age of 40, various disorders became visible in the superficial zone. The strict parallel orientation partially disintegrated, and tiny lesions appeared between the collagen fibers. Moreover, the arch-like orientation disappeared and a wavy collagen orientation was observed, characterized by strongly visible green fibers. This indicates the softening of the superficial zone, as evidenced by PLM scores of 3 (Fig. 5D). In the intermediate and deep zones, the maltan crosses were sometimes normally visible, while occasional abnormal crosses or shiny courts began to appear around the chondrocytes, exhibiting a PLM score of 3, and occasionally a score of 2 (Fig. 5D). In the intermediate and deep layers, the amount of thick and thin collagen fibers further decreased compared with young individuals (Figs. 4E and 5B, C). From the age of 51, the superficial layer of collagen orientation became increasingly disorganized and thicker. However, irregular shiny collagen bundles with wider gaps were observed (Fig. 4F). Additionally, some superficial discontinuity could occasionally be detected. Interestingly, from the age of 51 to 80, the thick collagen fibers became more prominent in the superficial layer (Fig. 4F-I). Although the thick fibers located parallel and longitudinally showed a stronger appearance, this orientation extended into the intermediate zone, losing its perpendicular appearance. Subsequently, the maltan crosses were not always detectable or their regular characteristics was modified in the intermediate zone (Fig. 4F-I). PLM score supported these data as superficial zone had PLM score 2–3, and intermediate zone showed PLM score of 3 (Fig. 5D). The ratio of thick and thin collagen fibers did not alter in the deep zone in the ages of 51 to 80 (Fig. 5B and C). In the age groups of 81 to 99, the superficial layer became thinner, and the parallel collagen fibers were sometimes not detectable in a consistent manner. Instead, diffuse signals became stronger or exhibited a green appearance (Fig. 4I and J). Additionally, a PLM score of 2 indicates the disorientation of cartilage (Fig. 5D). The maltan crosses in an individually variable manner became thinner and appeared with green court (Fig. 4I). The orientation was barely discernible, with a PLM score of 2 (Fig. 5D). The amount of both thick and thin collagen fibers was significantly reduced after the age of 90 (Figs. 4J and 5A-C), resulting in a PLM score of 2 in all zones (Fig. 5D).

### Expression of PACAP receptors during aging

**Fig. 6:**
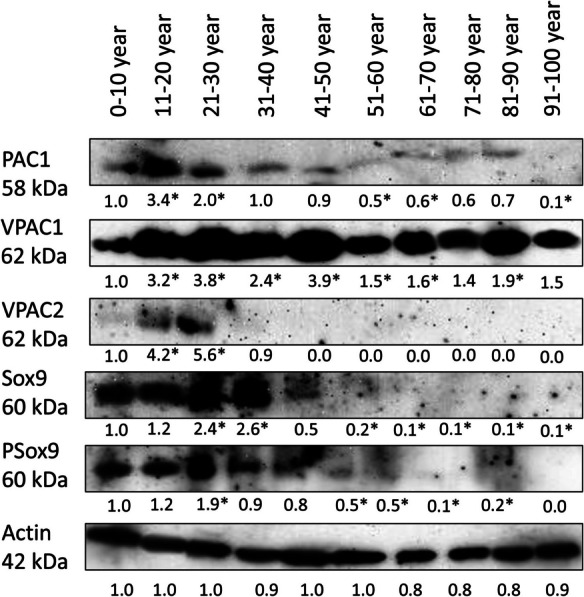
Investigation of PACAP and its receptors in articular cartilage. Protein expression of PAC1-R, VPAC1-R, VPAC2-R, Sox9 and P-Sox9 in articular cartilage. For Western blot reactions, actin was used as control. Optical signal density was measured, and results were normalized to the 0–10 age group. Numbers below signal panels represent integrated signal densities determined by ImageJ software. Asterisks indicate significant (*p < 0.05) alteration of expression compared to the respective control. Representative data of three independent experiments

Binding of PACAP to its receptors can trigger Sox9 activation; therefore, we followed their expression with Western blot during aging. Expression of the specific PACAP binding PAC1-R was detectable in articular cartilage. On the other hand, strong signals were visualized in young individuals with an increasing expression till the age of 30. Interestingly, between the age of 30 and 40, the expression of PAC1 receptor reduced to childhood level. After the age of 40, a slight reduction was detectable but almost a constant expression of PAC1 receptor was visualized till the age 90. The receptor was barely detectable in the last experimental group (Fig. 6). Expression of VPAC1-R appeared strongly in the articular cartilage. In childhood and in young adults, it showed an increase until the age of 50; then, a permanent expression was visible till the end of life (Fig. 6). The expression of VPAC2 receptor was detectable only in young individuals till the age of 30 but no signals were detected in the following decades (Fig. 6). Downstream target of PAC1 receptor can be Sox9, the expression of which showed a pattern similar to the receptor itself as it was visualized with a strong expression till the age of 50 with a strong elevation till the age of 40. After the age of 50, the detection of Sox9 was at the level of the limitation of Western blot technique, but with an extremely low level, it was present in articular cartilage (Fig. 6). Additionally, the phosphorylated, more active form of Sox9 showed a similar expression pattern to Sox9 and PAC1 receptor during aging (Fig. 6).

**Fig. 7:**
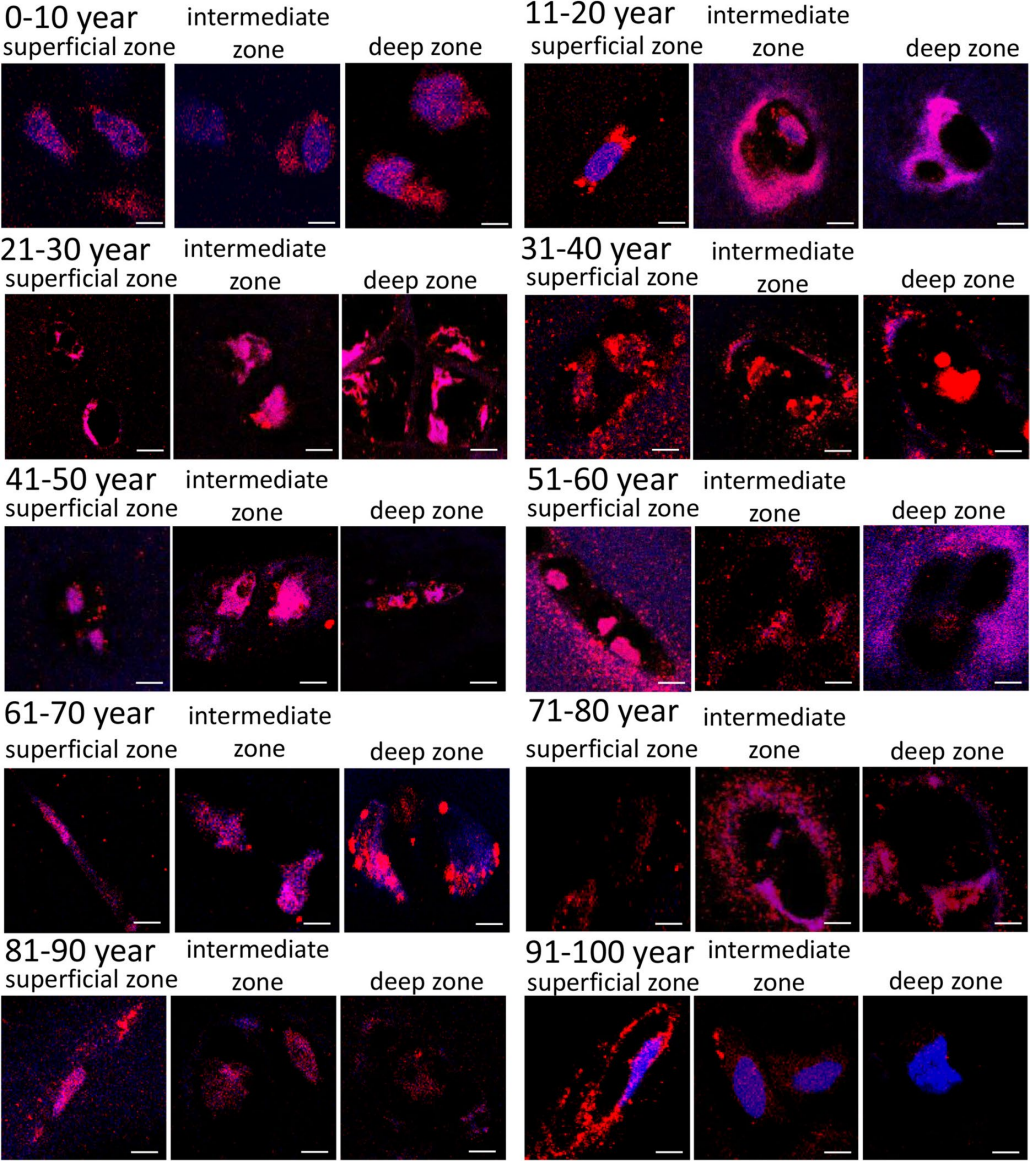
PAC1-R immunohistochemistry. Following groups of aging was set 0–10 age (**A**), 11–20 age (**B**), 21–30 age (**C**), 31–40 age (**D**), 41–50 age (**E**), 51–60 age (**F**), 61–70 age (**G**), 71–80 age (**H**), 81–90 age (**I**), and 91–100 (**J**). Original magnification was 60 ×. Scale bar: 5 µm. Representative data of 3 independent experiments

To verify the expression of PAC1-R, immunohistochemistry was performed. Expression of PAC1 receptor was detected in articular cartilage of children; in these samples, PAC1 receptor was located both intracellularly and intramembranously in superficial, intermediate, and deep zones. In young individuals, strong immunopositivity was detected in the membrane of chondrocytes in the superficial, intermediate, and deep layers till the age of 40 (Fig. 7). After the age of 41, the intracellular location of PAC1-R became more prominent although membrane signal also appeared in all layers of the articular cartilage. Although the immunopositive labeling of PAC1-R remained detectable in the superficial layer of cartilage, inconsistent membrane and cytosolic appearance alternately appeared till the end of life with a slight decrease (Fig. 7). Strange diffuse immunopositivity was present in various layers of cartilage after the age of 41. From the age of 81, the expression of the receptor became weaker in the intermediate and deep zones. PAC1-R immunopositivity in chondrocytes of the superficial layer was detected, showing a continuous reduction in the membrane of chondrocytes (Fig. 7). In contrast, the intermediate and deep zones of the joints exhibited decreased or slightly altered PAC1 receptor expression (Fig. 7).

## Discussion

**Fig. 8:**
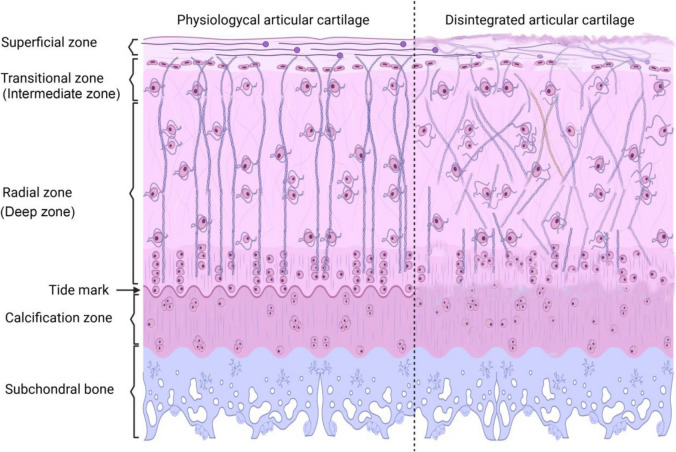
Structure of articular cartilage. Articular cartilage is organized into four distinct zones, each with unique structural and functional characteristics: Superficial zone: The outermost layer, composed of a thin film of collagen fibers arranged parallel to the articular surface. This orientation helps reduce friction during joint movement. Intermediate or transitional zone: Characterized by a higher concentration of proteoglycans, this zone has arch like arrangement of collagen fibers, providing the cartilage with elastic properties and resistance to compression. Deep or radial zone: This layer features thicker collagen fibers, aligned perpendicular to the articular surface, which provides strength and stability. It is crucial for withstanding the compressive forces experienced during weight-bearing activities. Calcification zone: The deepest layer, where the cartilage transitions to subchondral bone. It contains mineralized cartilage and helps anchor the cartilage to the underlying bone, facilitating the transfer of loads. The tide mark refers to a distinct boundary observed in articular cartilage that separates the uncalcified cartilage from the calcified cartilage layer. The left side represents the physiological orientation of collagen in articular cartilage, while the right part of the image illustrates the disintegration of collagen in articular cartilage. During aging, surface discontinuity and collagen disorientation can be observed, along with alterations in the columnar orientation of cells in the deep zones of articular cartilage. Additionally, the tide mark diminishes in aged cartilage

The knee joint carries the whole-body weight during locomotion. The hyaline cartilage covering the bones’ surfaces transmits this load to the more rigid bone tissue of the femur and tibia in an optimally attenuated manner. The articular cartilage, a type of hyaline cartilage, covers the end of major bones composing this joint and plays an important role in the maintenance of smooth sliding movement and mechanical stress response that transmits loads on the articular surface to the subchondral bone [44]. The highly elastic resistance of articular cartilage to the compressive forces is provided by the characteristic arrangement of ECM components and the high water content of the hyaline cartilage. Cartilage matrix is characterized by an abundance of type II collagen, hyaluronan, and the large chondroitin-sulfate rich proteoglycan, aggrecan (Fig. 8). These constituents play a crucial role in water retention and contribute to the integrity of the tissue [45]. All these components are produced and secreted by chondrocytes, the activity of which slows down in senescence. Since the tissue is avascular, the recovery or regeneration of extracellular matrix is very limited, especially in adulthood. The production of matrix components is definitely slower during aging, but age-dependent reduction of signalization has not been described in the cartilage in depth [46, 47]. Furthermore, it is also known that primary cilia of chondrocytes shows an age-dependent decrease affecting on mTOR signaling [48], and age-dependent decrease in PRG4 and BMPs of the temporomandibular cartilage has also been identified [49]. It is also known that FGF signaling alterations trigger age-dependent orthopedic disease formation [50].

PACAP and its correlating receptors, PAC1-R, VPAC1-R and VPAC2-R, were originally discovered as a peptide hormone system in the pituitary gland [24]. Later, its function and effects were shown in peripheral tissues throughout the body, including bone and cartilage tissues [24, 27]. PACAP is a positive regulator of in vitro chondrogenesis via signaling pathways that terminate, among others, in activation of Sox9 transcription factor. Sox9 is one of the essential signaling features of chondrogenesis inducing the expression of aggrecan and collagen type II [22]. On the other hand, the normal expression and function of Sox9 postnatally are essential for maintaining the integrity of the cartilage matrix, which can help prevent the formation of OA. Additionally, Sox9 continuous expression can keep the growth plates open [51]. Although this transcription factor is a key regulator in matrix production, its detailed expression has not been investigated in aging processes of human articular cartilage in detail. The goal of our research was to examine the aging of human knee cartilage samples and to find a correlation with the expression levels of receptors signaling for PACAP neuropeptide, along with other parameters that indicate degeneration of the cartilage tissue. Previous studies indicated that PACAP levels were negatively associated with the extent of meniscus injuries [52]. Patients with higher synovial fluid PACAP levels presented less cartilage lesions. Moreover, PAC1-R expression increase was demonstrated in osteoarthritic knee joints [31]. The presence of PACAP is crucial for proper cartilage formation, and the chondro-protective effect of PACAP has also been demonstrated [22, 28]. The expression of the PAC1 receptor protein was identified in chondroprogenitor cells, and PACAP could promote extracellular matrix synthesis indicating the positive effect of PACAP in cartilage development [53]. In addition, other studies showed a weak or even absent PACAP expression in OA cartilage, correlating with reduced cellularity of the cartilage [31]. The changes in PACAP expression in response to inflammation were associated with increasing the defiance against cell death stimulated by IL1-β in primary chondrocyte cultures [31]. Our previous experiments on knee articular cartilage from PACAP KO mice revealed early aging characteristics in mice deficient in endogenous PACAP, compared to WT mice. There was a remarkable difference in PAC1-R expression, which was predominantly higher in WT mice and a profound decrease in aged PACAP KO mice [30].

To follow the expression level of the PACAP receptors (PAC1-R, VPAC1-R, VPAC2-R) along human aging, we divided the samples into decades. Our results demonstrated reduction in cartilage thickness during aging together with reduction of the cartilage integrity, specifically in the glycosaminoglycan content. Based on OARSI pathological scoring, we established that during aging, the prevalence of cartilage destruction is significantly increased, particularly from the 4 th decade of life. In these ages, the orientation of collagen fibers, their thickness parallel with the secreted GAGs content started to show disorders in human articular cartilage. These results indicate that physical injuries and mechanical forces can result in softening, subsequently in the formation of OA in the 40 s with a higher risk. Similar results have been published in meniscus repair, where the threshold of successful treatment was set at the age of 40 [54]. Additionally, there are some clinical studies which demonstrated the age dependency of cartilage regeneration where the 4 th decade of life is fundamental in successful medical treatment [55]. Furthermore, some data show that formation of OA after anterior cruciate ligament injury is likely after the age of 40 [56]. The reason for the formation of chondropathies is usually the disintegrated collagen type II and aggrecan characteristics (Fig. 8), which can be due to decreased Sox9 activity [57]. To connect those findings with the variability of PACAP receptors during aging, we showed a correlation between the expression levels of PAC1-R and the transcription factor Sox9, as well as other findings related to cartilage degradation and reduction in thickness. Based on our findings, the expression of PAC1-R can predict how chondrocytes can counteract and regenerate in response to various mechanical and chemical stresses [29, 58]. Cells that express PACAP receptor subtypes are able to respond to the neurotransmitter stimuli in a positive feedback that promotes the expression of more cell surface receptors to intensify the stimuli. Based on previous investigations in the field, we can suggest that PACAP has a crucial role in cartilage integrity maintenance and cartilage regeneration. During aging, there is a significant reduction of PAC1-R in chondrocytes, along with the appearance of aging characteristics of OA and tissue disintegration (Fig. 8). The difference in the expression of PAC1-R by chondrocytes reflects differences between healthy and pathological cartilage affected by OA. As aging progresses, the level of PAC1-R expression declines in parallel with the chondrocytes’ ability to maintain the ECM structure in response to mechanical or chemical stress applied to the surrounding environment.

Interestingly, the intracellular location of PAC1-R was also detected in various zones of cartilage, as it has already been demonstrated in the testis [59]. Although the function of intracellularly flipped PAC1-R is not fully understood, it may serve as an internal reservoir for the later activation of this signaling through an endosomal mechanism during aging [60]. Alternatively, it can be located on rough endoplasmic reticulum [61] for modifying posttranscriptional processes, as it was demonstrated in the central nervous system. In other experiments, it has been shown the PAC1 receptor at 37 °C predominantly stayed in the cell membrane. However, after just a few minutes of exposure to nanomolar PACAP concentrations, there was significant endocytosis of PAC1-R into intracellular vesicles, accompanied by a noticeable decrease in cell surface immunopositivity. In contrast, similar PACAP exposures at 22–24 °C led to minimal receptor movement into intracellular vesicles and no reduction in membrane positivity, suggesting that receptor internalization was inhibited at room temperature [62]. In articular cartilage, due to the low oxygen tension, metabolism of chondrocytes is anaerobic which causes an acidic pH. Moreover, upon mechanical load, the temperature of articular cartilage increases. Therefore, it is likely that intracellular localization of the PAC1 receptor can be modified by the pH and temperature alterations under either normal locomotion or when inflammation occurs in an OA joint. The degradation of PACAP receptors during aging can be the consequence of decreased PACAP concentration, subsequently its reduced activation. Similar findings have been demonstrated in Parkinson’s disease model where decreased PACAP level resulted in a lower expression of PAC1 receptor [63]. Aging is often associated with changes in gene expression regulation, which could be mediated by epigenetic modifications such as DNA methylation, histone modifications, or microRNA dysregulation. These modifications can alter the expression of genes involved in receptor synthesis, potentially reducing PAC1 receptor expression. For example, age-related DNA methylation in the promoter region of the PAC1 receptor gene could lead to decreased transcription. Similar findings have been demonstrated in PTSD syndrome where the methylation of PAC1 receptor altered its expression [64]. The role of microRNAs that target PAC1 receptor mRNA could also contribute to its downregulation during aging although no published data can be found. Moreover, peak expression of VPAC1 and VPAC2 receptors can also be identified in young adults, supporting the multiple activation of PACAP signaling. Similarly to our results, it has been demonstrated that VPAC receptors do not always show reduction in aged animals [65].

Additionally, reactivating the PAC1 receptor through PACAP administration or other factors, such as oxidative stress or mechanical load, may modify its expression and contribute to the prevention of OA formation. There are some studies where it was found that blue light and ROS, specifically hydrogen peroxide, induced dominant nuclear translocation of PAC1-R, enhancing its promoter activity and protein expression. There are some inhibitors that reduced PAC1-R nuclear translocation and its subsequent positive feedback on PAC1-R expression [66]. Other studies have shown that PACAP 1–38, along with cellular oxidative stress, induces the nuclear translocation of PAC1-R and upregulates PAC1-R expression in a manner that is positively correlated with its nuclear translocation [67].

Cartilage health is crucial for maintaining a healthy life span. Aging, or senescence, of cells in articular cartilage partly depends on the integrity of the ECM (Fig. 8). This suggests that cell adhesion to the ECM, which is involved in characteristic morphological changes, may play a pivotal role in cellular senescence [68]. The mechano-biological properties of cartilage show age dependence, and decrease of lubrication of articular cartilage elevates the risk of OA formation [69]. Recent treatments have focused on siRNA application where the decreased activity of MMPs and ADAMTs is suggested to be the most effective way of OA curability [70]. It is worth noting that the activity of these matrix degrading enzymes is partly inhibited by PACAP signaling as we have demonstrated earlier [28]. The understanding of the components that contribute to this process is necessary for the development of medical applications that can restore or suppress the progression of OA, which is often unavoidable. The previous knowledge obtained concerning the mechanism by which PACAP and its receptors on chondrocytes contribute to the process of cellular defense and restoration against various stresses that have been continuously applied to the articular cartilage [71]. The conclusion gained from our study is that the expression of PAC1-R decreases in parallel with the reduced ability of the cartilage to maintain tissue integrity. This reduction in ability results in recognizable progressive cartilage deterioration. In addition to the observation that PACAP signaling in certain cartilage tissues decreases with aging, this implies that increasing the concentration of PACAP within the cartilage ECM may help counteract the upregulation of PAC1 receptors on cell surfaces. This process could involve the artificial stimulation of PAC1-R by administering a modulated solution directly to the joint capsule. This solution would contain a recombinant PACAP peptide to restore and maintain the integrity of the tissue in cases of OA.

## Supplementary Information

Below is the link to the electronic supplementary material.

Fig. 9 Supplementary Figure 1

High resolution image (TIF 78 KB)

Fig. 10 Supplementary Figure 2

High resolution image (TIF 122 KB)

Fig. 11 Supplementary Figure 3a

High resolution image (TIF 82 KB)

Fig. 12 Supplementary Figure 3b

High resolution image (TIF 82 KB)

## Abbreviations

AC: Adenylate cyclase
ADAMT: A disintegrin and metalloproteinase with thrombospondin motifs
ATP: Adenosine triphosphate
BMP: Bone morphogenetic protein
BSA: Bovine serum albumin
cAMP: Cyclic adenosine monophosphate
CREB: CAMP response element-binding protein
DAPI: 4′,6-Diamidino-2-phenylindole
DMMB: Dimethyl methylene blue
DPX: Dibutyl phthalate and xylene
ECM: Extracellular matrix
EDTA: Ethylene diamine tetra-acetic acid
EGTA: Ethylene glycol-bis(β-aminoethyl ether)-N,N,N′,N′-tetraacetic acid
ERK: Extracellular signal-regulated kinase
FGF: Fibroblast growth factor
GAG: Glycosaminoglycan
HE: Hematoxylin and eosin
KO: Knockout
mTOR: Mammalian target of rapamycin
NA: Numerical aperture
NMDA: N-methyl-D-aspartate
OA: Osteoarthritis
OARSI: Osteoarthritis Research Society International
PAC1-R: Pituitary adenylate cyclase-activating polypeptide type I receptor
PACAP: Pituitary adenylate cyclase activating polypeptide
PBS: Phosphate buffered saline
PBST: Phosphate buffered saline supplemented with 1% Tween-20
PIC: Protease inhibitor cocktail
PLM: Polarized light microscopy
PMSF: Phenylmethylsulfonyl fluoride
PKA: Protein kinase A
PRG4: Proteoglycan 4
SDS-PAGE: Sodium dodecyl sulfate–polyacrylamide gel electrophoresis
siRNA: Small interfering RNA
Sox: SRY-related HMG-BOX gene
TRPV: Transient receptor potential vanilloid
VIP: Vasoactive intestinal peptide
VPAC: Vasoactive intestinal peptide receptor
WT: Wild type
