# Supplementary figures and images for: Cartilage degradation is followed by PAC1 receptor reduction in articular cartilage of human knee joints
Source: GeroScience. 2025 May 14;48(1):915–36. doi: 10.1007/s11357-025-01689-4 (PMC12972452; doi:10.1007/s11357-025-01689-4)

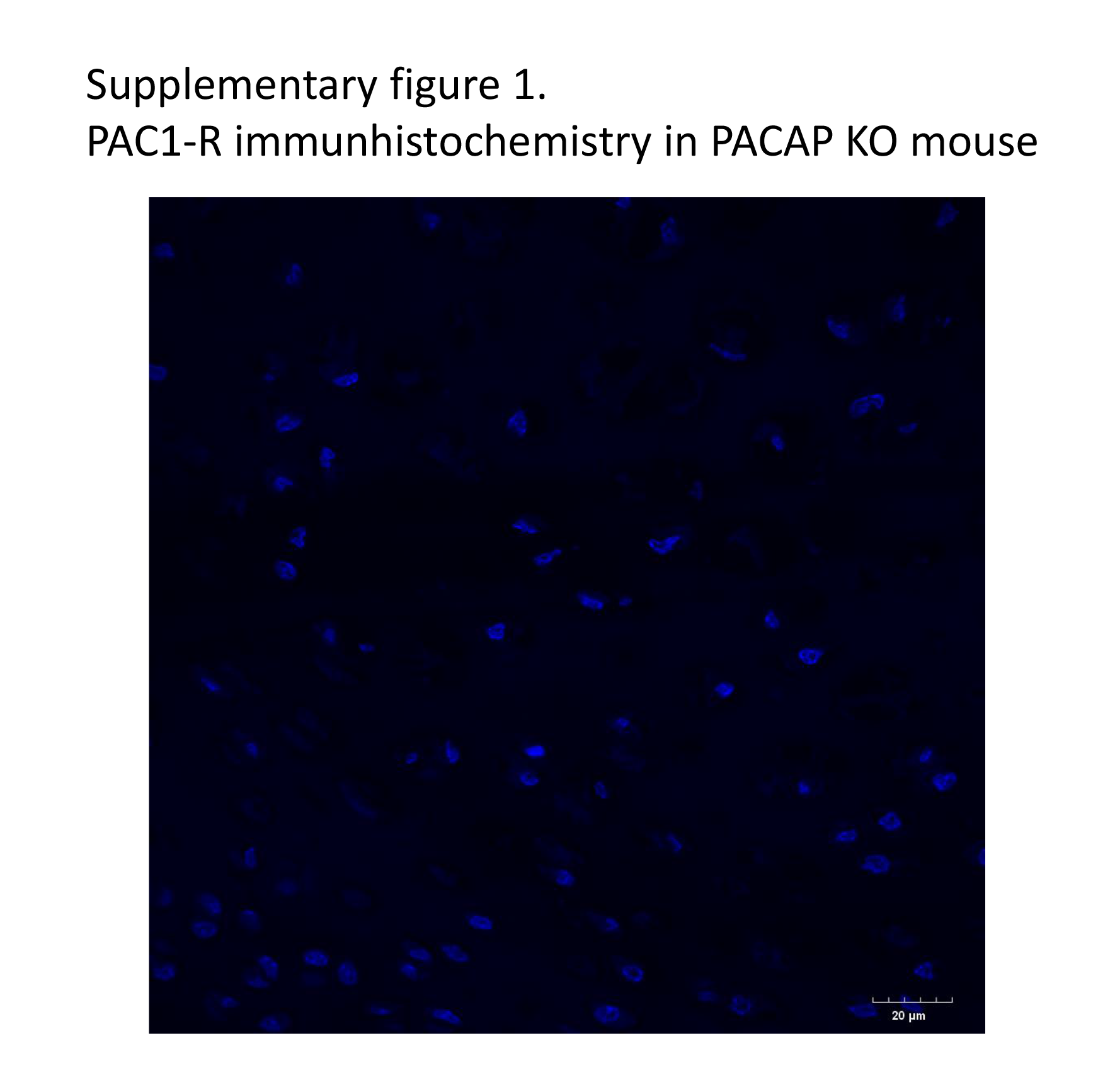

Supplement: Supplementary file 1 — Supplementary Figure 1 [file 11357_2025_1689_Fig9_ESM.png]

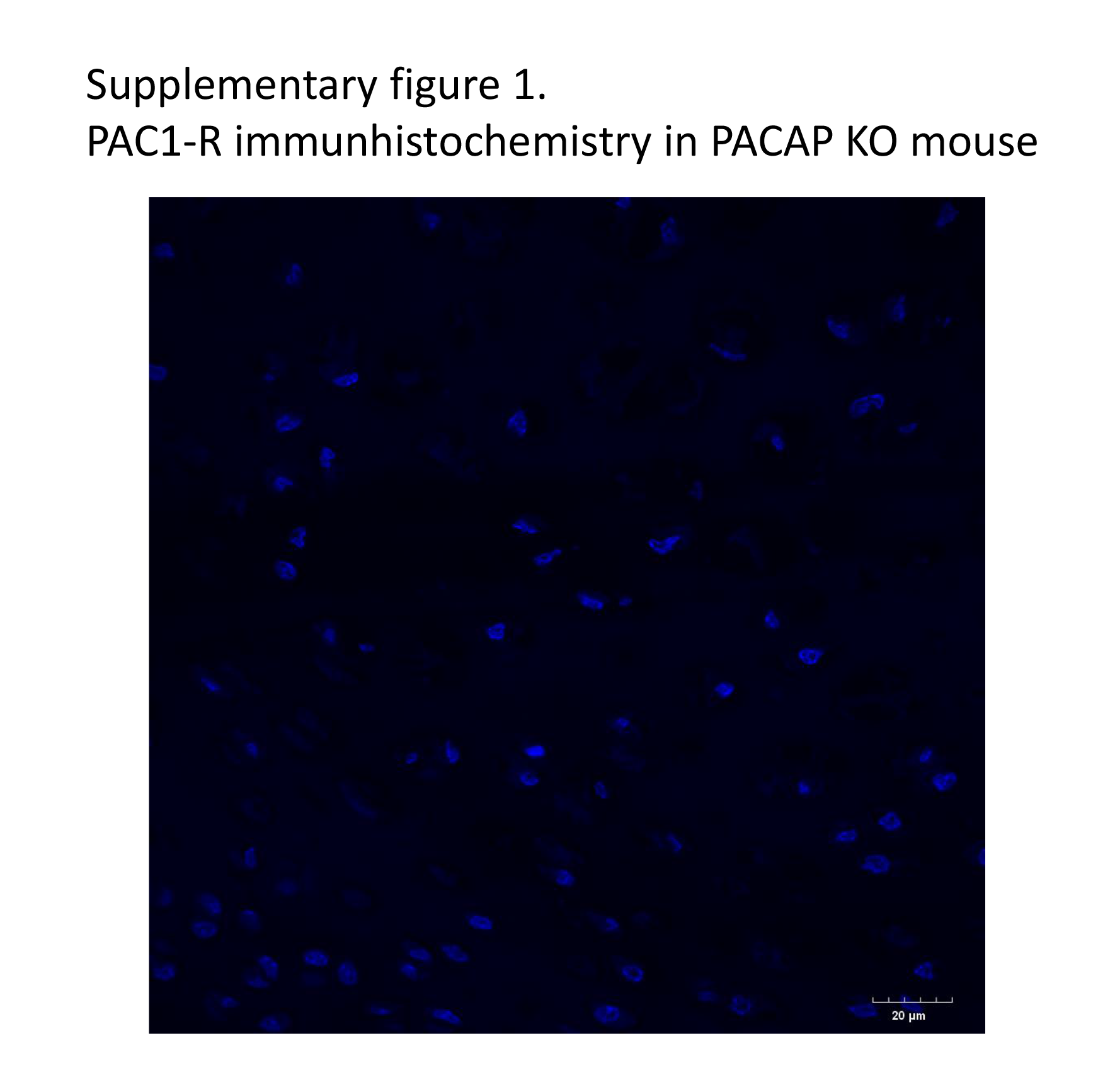

Supplement: Supplementary file 2 — High resolution image (TIF 78 KB) [file 11357_2025_1689_MOESM1_ESM.tif]

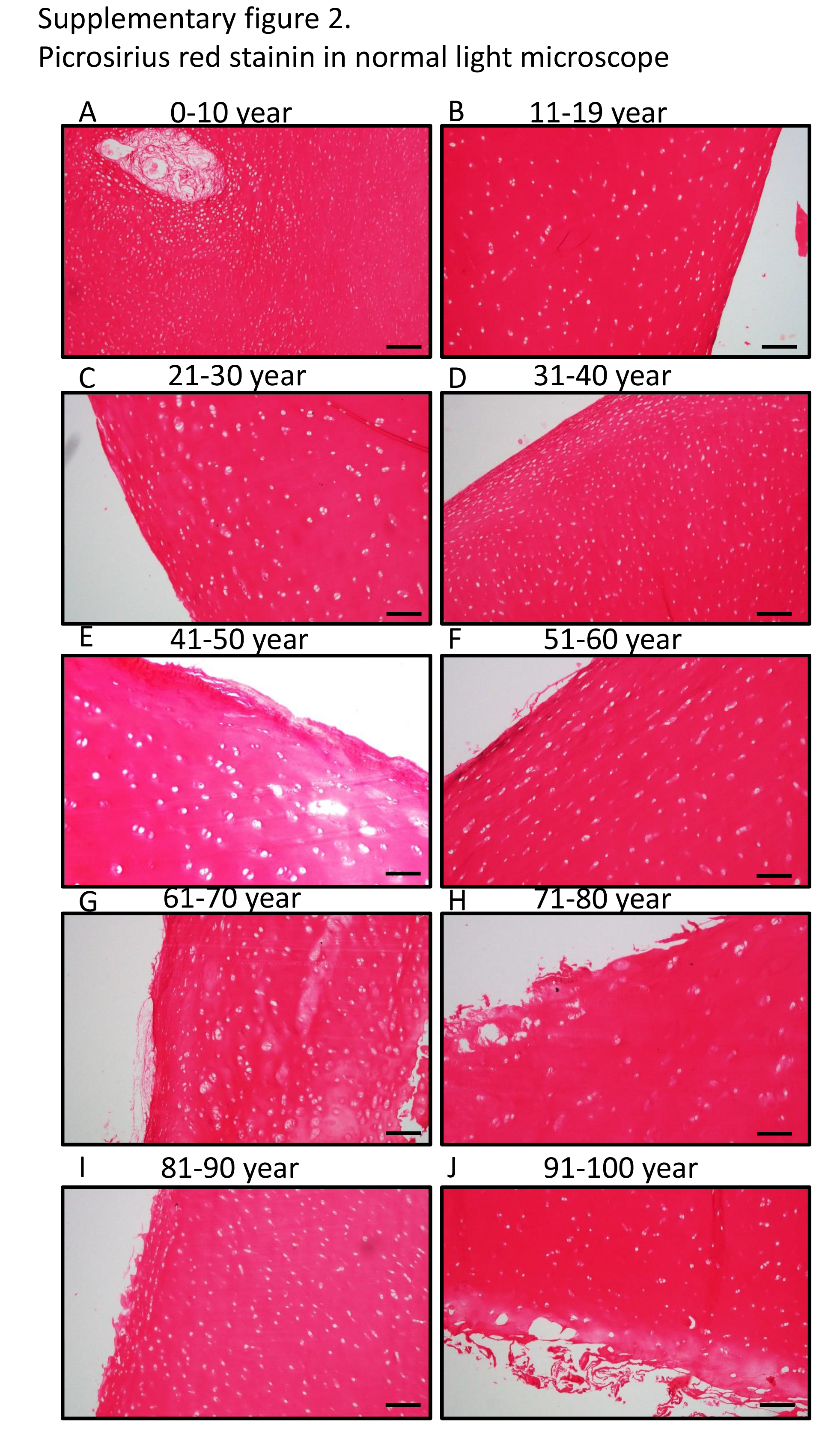

Supplement: Supplementary file 3 — Supplementary Figure 2 [file 11357_2025_1689_Fig10_ESM.png]

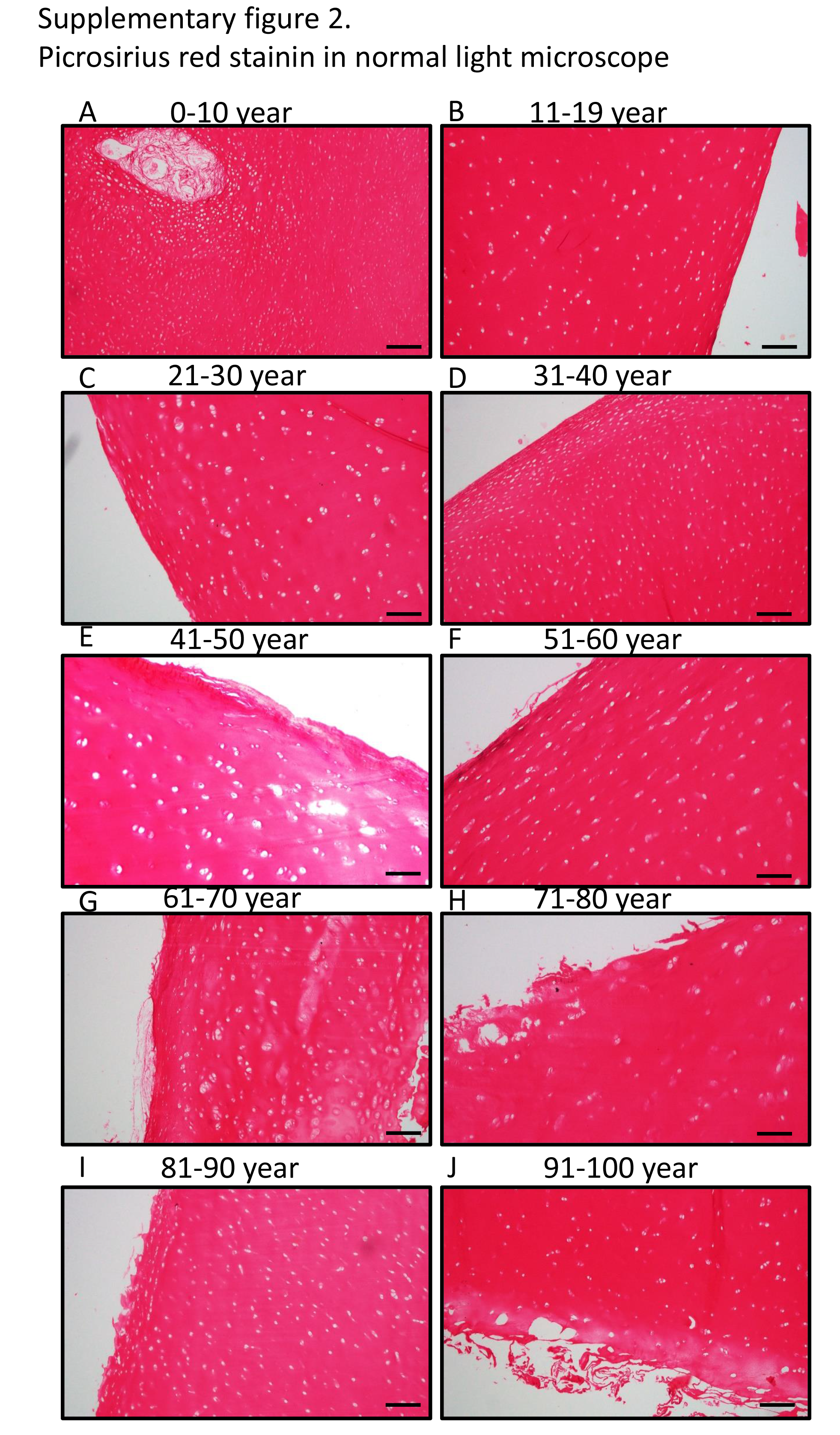

Supplement: Supplementary file 4 — High resolution image (TIF 122 KB) [file 11357_2025_1689_MOESM2_ESM.tif]

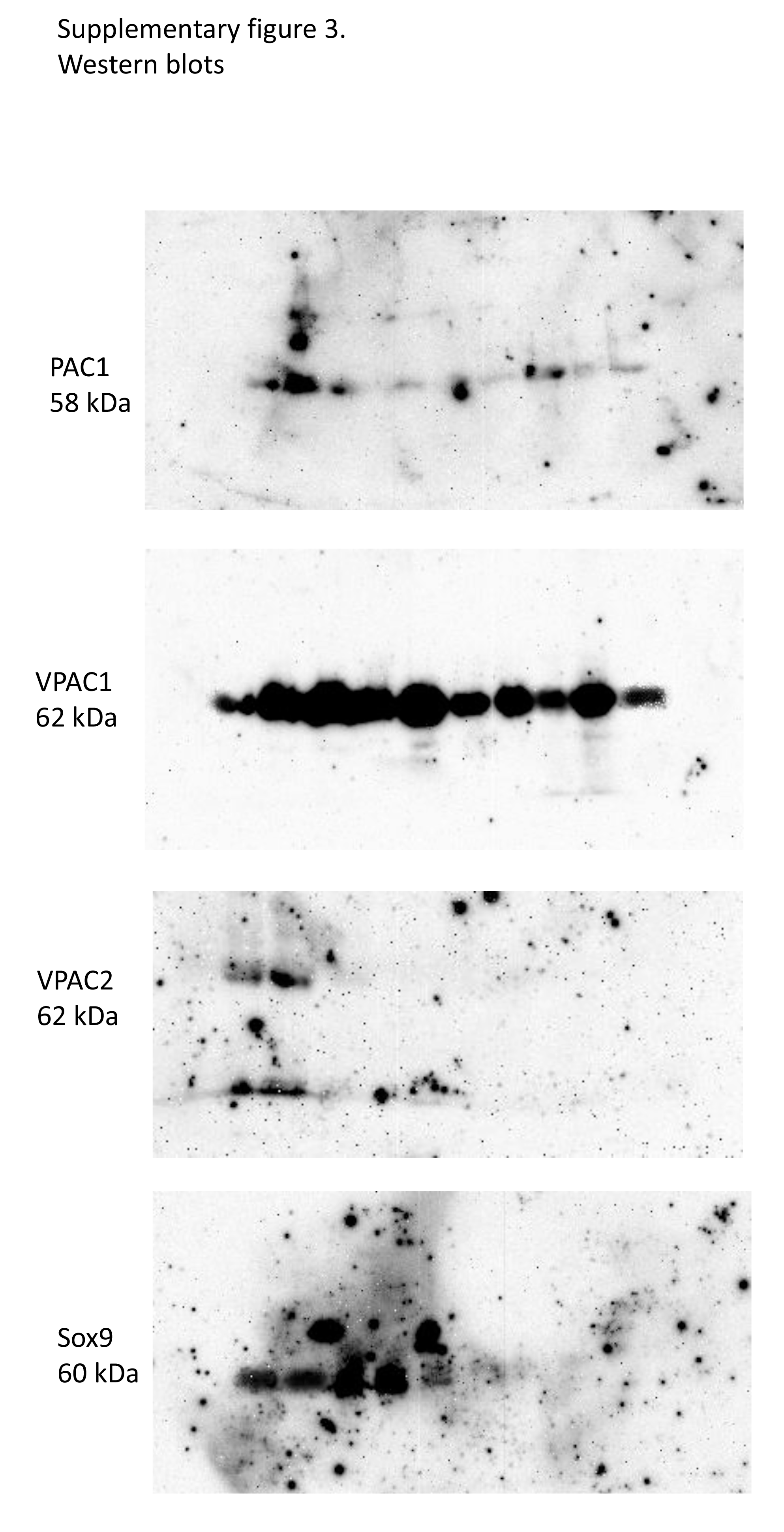

Supplement: Supplementary file 5 — Supplementary Figure 3a [file 11357_2025_1689_Fig11_ESM.png]

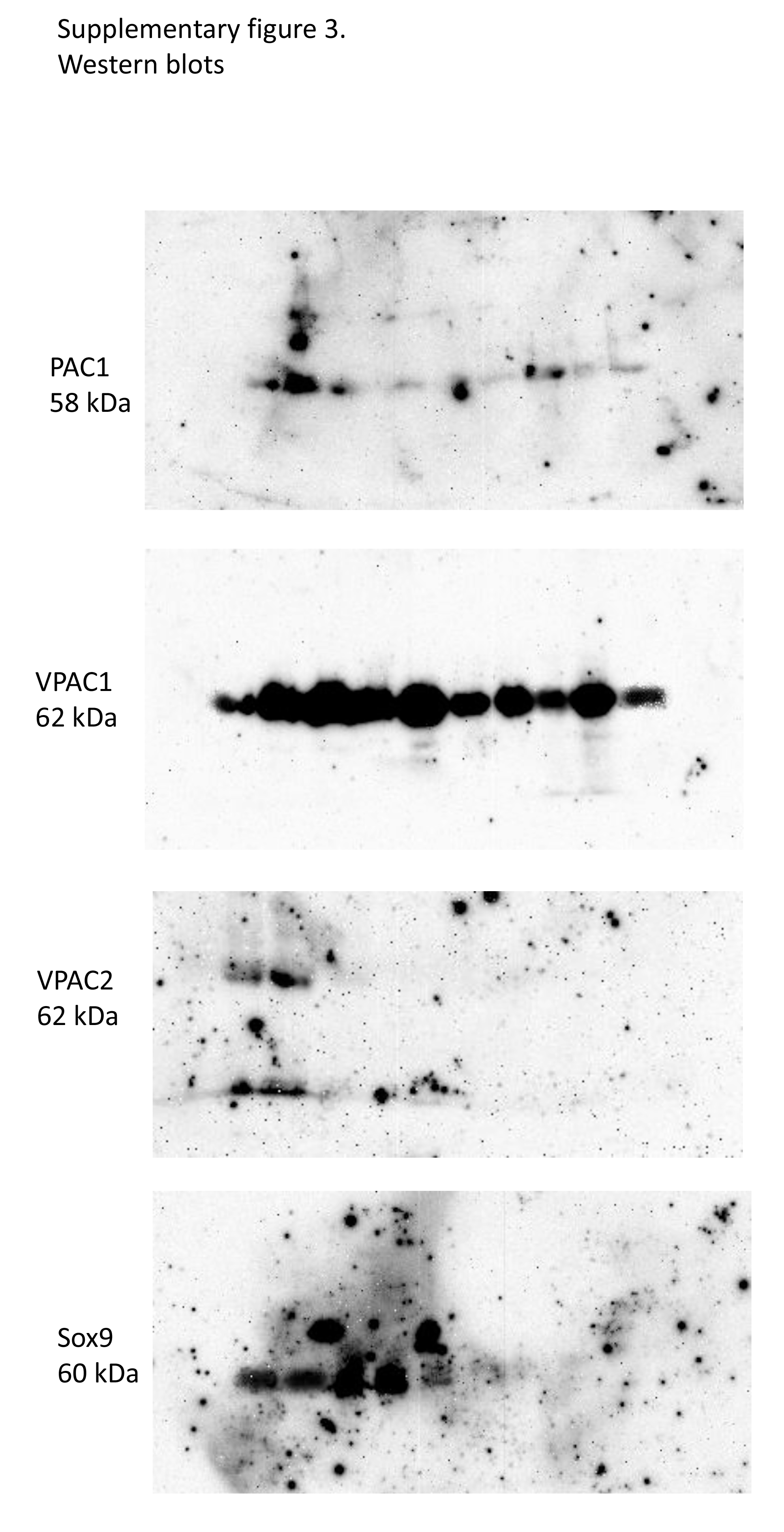

Supplement: Supplementary file 6 — High resolution image (TIF 82 KB) [file 11357_2025_1689_MOESM3_ESM.tif]

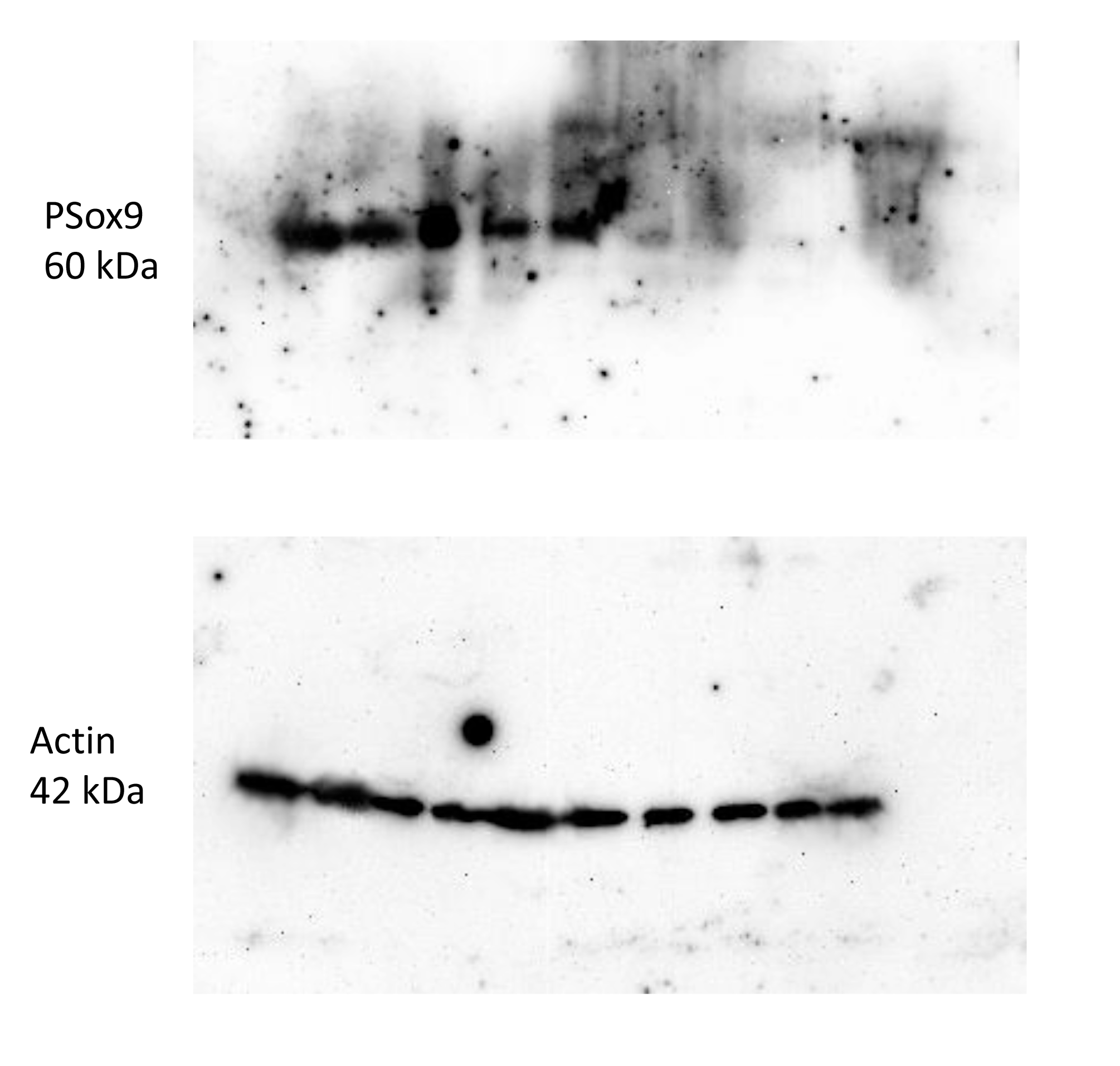

Supplement: Supplementary file 7 — Supplementary Figure 3b [file 11357_2025_1689_Fig12_ESM.png]

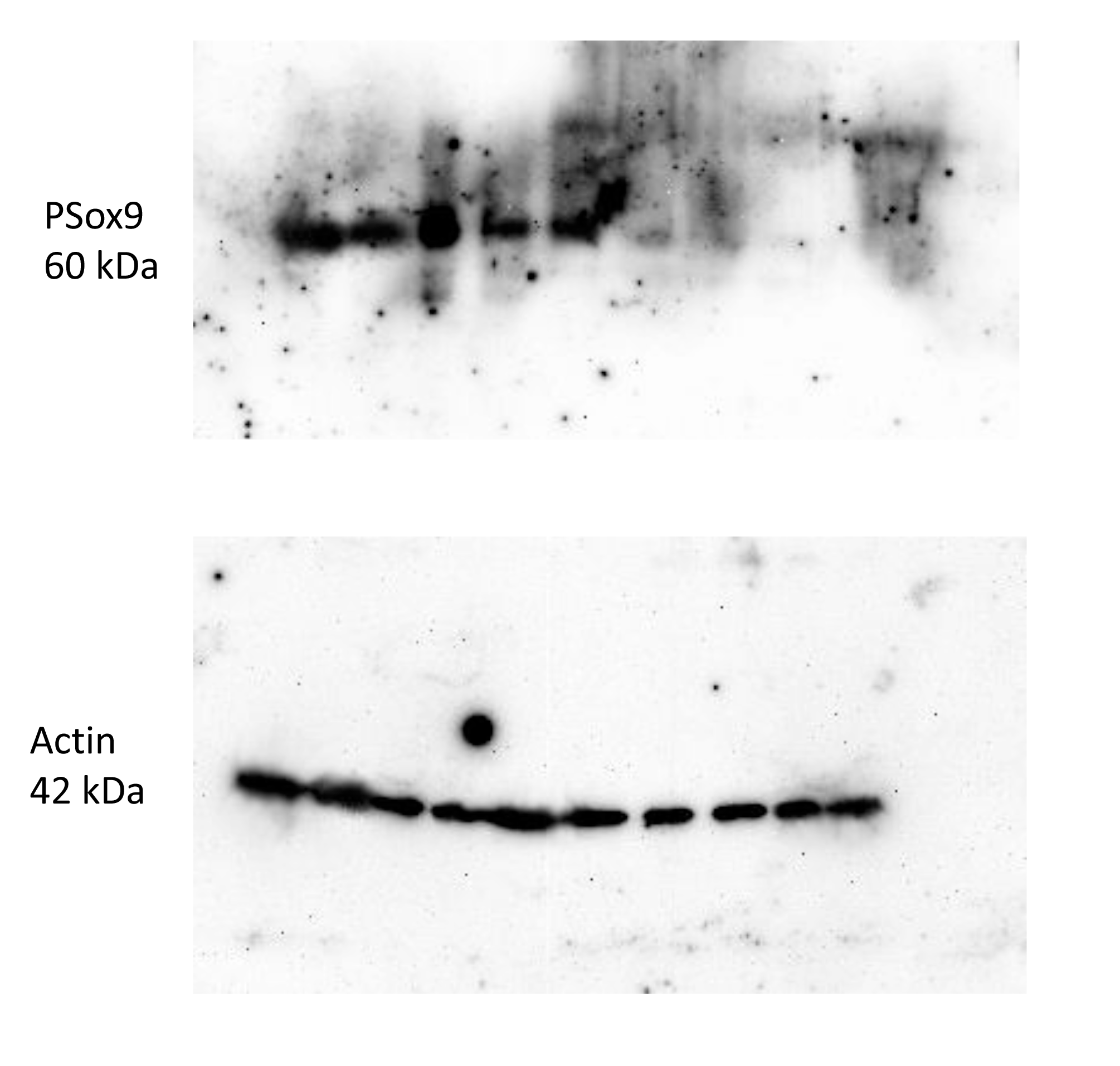

Supplement: Supplementary file 8 — High resolution image (TIF 82 KB) [file 11357_2025_1689_MOESM4_ESM.tif]
